# Findings from the Indonesian family life survey on patterns and factors associated with multimorbidity

**DOI:** 10.1038/s41598-023-42603-2

**Published:** 2023-10-30

**Authors:** Meliana Griselda, Sofa D. Alfian, Imam A. Wicaksono, Martin Wawruch, Rizky Abdulah

**Affiliations:** 1 Department of Pharmacology and Clinical Pharmacy, Faculty of Pharmacy, Universitas Padjadjaran, Jl. Raya Jatinangor, KM 21, Jatinangor, Sumedang Indonesia; 2 Pharmacist Profession, Faculty of Pharmacy, Universitas Padjadjaran, Jatinangor, Indonesia; 3 Drug Utilization and Pharmacoepidemiology Research Group, Centre of Excellence for Pharmaceutical Care Innovation, Universitas Padjadjaran, Jatinangor, Indonesia; 4 Center for Health Technology Assessment, Universitas Padjadjaran, Jatinangor, Indonesia; 5 Institute of Pharmacology and Clinical Pharmacology, Faculty of Medicine, Comenius University, Bratislava, Slovakia

**Keywords:** Risk factors, Cardiovascular diseases

## Abstract

The prevalence of multimorbidity tends to increase with age, but it is now also reported in the middle-aged population, which has a negative impact on healthcare systems and health outcomes. This study aims to analyze the patterns and factors associated with multimorbidity in Indonesia. This national cross-sectional population-based survey used publicly available data from the Indonesian Family Life Survey (IFLS-5) for 2014 among middle-aged (40–59 years old) and elderly (≥ 60 years old) respondents. Information on all chronic diseases was assessed using a self-reported questionnaire. Sociodemographic and health-related behavioral factors were obtained from self-reported data. Binary logistic regression analysis was used to identify the factors associated with multimorbidity. Adjusted odds ratios (AORs) with 95% confidence intervals (CIs) were reported. The study recruited 11,867 respondents. The prevalence of multimorbidity was 18.6% (95% CI 17.9–19.3) with which 15.6% among middle age (95% CI 14.95–16.25) and 24.9% among the elderly (95% CI 24.12–25.68). Hypertension was the most commonly reported disease (23.2%) in all combinations of multimorbidity and among all age groups. Socio-demographic factors: elderly (AOR: 1.66; 95% CI 1.46–1.89), female (AOR: 1.42; 95% CI 1.20–1.69), living in the urban area (AOR: 1.22; 95% CI 1.09–1.38), higher educational level (AOR: 2.49; 95% CI 1.91–3.26), unemployed (AOR: 1.63; 95% CI 1.44–1.84), and higher economic level (AOR: 1.41; 95% CI 1.18–1.68) were associated with multimorbidity. Poor health behavior factors: being former smokers (AOR: 2.03; 95% CI 1.65–2.51) and obesity (AOR: 1.53; 95% CI 1.35–1.75) were also associated with multimorbidity. The prevalence of multimorbidity in the middle-aged and elderly population in Indonesia is relatively high, particularly in populations with poor health behaviors. Therefore, healthcare professionals should integrate more patient-specific factors when designing and implementing tailored interventions to manage multimorbidity in Indonesia.

## Introduction

Noncommunicable diseases have risen to seven of the top ten causes of death worldwide because chronic diseases account for up to 74% of all deaths globally^1^. While chronic disease was the leading cause of death in Indonesia in 2016, accounting for 73% of all deaths^2^, its prevalence has increased with age—an increase observed in both the elderly and middle-aged populations^3,4^. The elderly population is predicted to increase globally to reach 1.5 billion people^5^, meanwhile, chronic illness starts to develop due to physiological changes in the middle-aged population.

Multimorbidity, or the simultaneous existence of two or more chronic diseases, has a significant impact on health status and requires complex healthcare^6^. Multimorbidity results in a more complex adverse impact on health and economic status, such as accrual healthcare use and expenditure^7^, which can lead to increased social and healthcare needs^8^. Simultaneously, the risk of polypharmacy and patient management has increased and become more complex. However, the resources needed to manage the problems are limited^9^, especially in low- and low-middle-income countries. Although multimorbidity is commonly defined as the presence of two or more chronic diseases, it also implies that these conditions are not a hierarchical dependence on each other. The concept was created to substitute the idea of co-morbidity, emphasizing the importance of addressing all patients' health conditions^10^. A consequence of this shift towards the concept of multimorbidity is that the pattern in which these conditions appear is now fundamental to tailor the treatment to the patient's needs and to better match the health professional's skills with the patient's diseases^11,12^. Nevertheless, multimorbidity can be prevented by targeting and managing factors associated with multimorbidity in health interventions.

In Indonesia, the prevalence of multimorbidity among the population aged 40 years old and above was reported to be at least more than 20% and showed an increment as the population was older^3,13–15^. A previous study reported that 44.4% of the elderly population in Indonesia have multimorbidity^16^. However, the prevalence of multimorbidity is now also reported in the middle-aged population (age range of 40–59 years)^17^. Previous cross-sectional studies reported that approximately 30–50% of middle-aged patients experienced multimorbidity. The absolute number of such patients may even exceed the number of patients with multimorbidity in the elderly^3,4^. A systematic review that used data from 2000 to 2021 showed the prevalence of multimorbidity among the middle-aged population had reached more than 45% globally^18^. It is also stated that the middle-aged population in Indonesia was more likely to have multimorbidity than the younger generations^13^.

Previous studies in Indonesia have assessed factors that affect multimorbidity among the Indonesian population^13–16,19,20^. Most of these studies, however, included limited potential factors associated with multimorbidity and only included the elderly and/or mixed population. On the other hand, there may be another factor that needs to be addressed to acknowledge their association with multimorbidity. Furthermore, compared to the elderly population, the middle-aged population has received less attention regarding multimorbidity development. It is possible that the middle age population has additional distinct factors associated with the development of multimorbidity^21^*.*

Therefore, in this study, we aimed at describing pattern and factors associated with multimorbidity among middle-aged to elderly individuals in Indonesia.

## Methods

This study was reported according to the Strengthening the Reporting of Observational Studies in Epidemiology (STROBE) guidelines for cross-sectional studies^22^ [See additional file 1: Table S1, Supplementary data].

### Study design and data source

We conducted an observational cross-sectional study design based on the fifth wave of the national longitudinal data of the Indonesian Family Life Survey (IFLS-5), which were collected from 2014 to 2015, and data have been publicly available since 2016 (https://www.rand.org/well-being/social-and-behavioral-policy/data/FLS/IFLS/access.html). IFLS-5 is an ongoing health and socioeconomic survey on the individual, household, and community levels that had been implemented by the RAND Corporation since 1993^23^. IFLS-5 uses a multistage stratified sampling design that covers 83% of the Indonesian population and is conducted in thirteen provinces, including four provinces on Sumatra (North Sumatra, West Sumatra, South Sumatra, and Lampung), all five of the Javanese provinces (DKI Jakarta, West Java, Central Java, DI Yogyakarta, and East Java), and four provinces covering the remaining major island groups (Bali, West Nusa Tenggara, South Kalimantan, and South Sulawesi), with a response rate of more than 90%^23^. These respondents were followed in subsequent waves that were held in 1997, 2000, 2007, and 2014^23^.

### Study population

Data were obtained from respondents who were at least 40 years of age at the completion of the survey. Respondents without available data on chronic diseases were excluded.

### Outcome measures

Multimorbidity is defined as the presence of two or more chronic conditions in an individual at the same time^6^. Multimorbidity was assessed through a self-report questionnaire with the question, “Has a doctor/paramedic/nurse/midwife ever told you that you had the following chronic conditions of disease?,” with response options: hypertension, diabetes, asthma, chronic lung diseases, cardiac diseases (heart attack/coronary heart disease/angina or other heart diseases), liver diseases, stroke, cancer or malignancies, arthritis/rheumatism, uric acid/gout, hypercholesterolemia, prostate illness, kidney diseases, stomach or other digestive diseases, psychiatric problems, and memory-related diseases^23^.

### Potential factors associated with multimorbidity

Sociodemographic factors were assessed based on a self-reported questionnaire and included age (40–59 years old/above 60 years old), gender (male/female), household location (urban/rural), level of education (no formal education or kindergarten, elementary school, middle school, high school, university), working status in the last 12 months (working/not working), marital status (currently married, currently not married, including unmarried or single (has not married yet), separated (no longer living together) or divorced (divorced legally) and widow(er) (lost one's spouse through death), economic status (per-capita expenditure for consumption which included monthly expenditure for the following food items [rice, meat and fish, vegetables, cooking oil, and granulated sugar]), and non-food items (kerosene)^14^. This per-capita monthly expenditure was categorized into quintiles: Q-1 (lowest) to Q-5 (highest)^24^.

Health-related behavioral factors were assessed through a self-reported questionnaire, including smoking habit (non-smoker, former smoker, current smoker), frequency of fruits and vegetable consumption (at least 7 days per week/4–6 days per week/less than 4 days per week)^15^, Body Mass Index (BMI) which was calculated based on the self-reported weight and height of the respondents and then was categorized into 4 groups (underweight [< 18.5 kg/m^2^], normal [18.5–22.9 kg/m^2^], overweight [23–24.9 kg/m^2^], and obese [≥ 25 kg/m^2^])^25^, and physical activity. Physical activity was assessed through the metabolic equivalent of task (MET), that is, multiples of the resting metabolic rate. These MET scores were then multiplied by the minutes performed^26^. MET-minutes/week score was obtained through specific calculations based on the International Physical Activity Questionnaire (IPAQ) guidelines and then classified into three categories: less active (MET-minutes/week less than 600), moderate (MET-minutes/week in the range of 600–2999), and highly active (MET-minutes/week equal to 3000 or above for combination of those three types of physical activity, or equal to 1500 or above for vigorous type of physical activity)^26^.

### Statistical analysis

The characteristics of the respondents were analyzed through descriptive analysis. We used Little's Missing Completely at Random (MCAR) test to determine the type of missing data. In this test, the null hypothesis is that the data being tested are MCAR. If the significance value of the test results exceeds the p-value (> 0.05), the MCAR assumption is met, implying that no correlation exists between the pattern of missing data and the existing data in the analysis^27^. The proportion of missing data that is less than 10% ± 5% will usually not cause any biases in a study^28^.

The frequency of the combinations of chronic diseases was carried out by counting the most occurring pairs and triplets of diseases. The pairing results are then summed for each group and then sorted from the highest to the lowest frequencies. A chi-square test was performed to assess the bivariate association between the characteristics of the respondents and outcomes. The potential factors, which were found to be associated with the outcome at a significance level of p < 0.25 in the bivariate analyses [See additional file 1: Table S2, Supplementary data], were included in the initial multivariate model^29,30^. The variance inflation factor (VIF) was further analyzed to eliminate multicollinearity. Multivariate binary logistic regression was performed to obtain the adjusted odds ratio (AOR) with a 95% confidence interval (95% CI) with manual backward elimination. The p-values were set at 0.05 for the factors included in the final model. Subgroup analysis by age group was carried out. Omnibus and Nagelkerke R-Square tests were used to assess the multivariate model. All statistical analyses were performed using the Statistical Package for the Social Sciences (SPSS) version 26.0.

## Results

### Respondent’s characteristics

**Figure 1:**
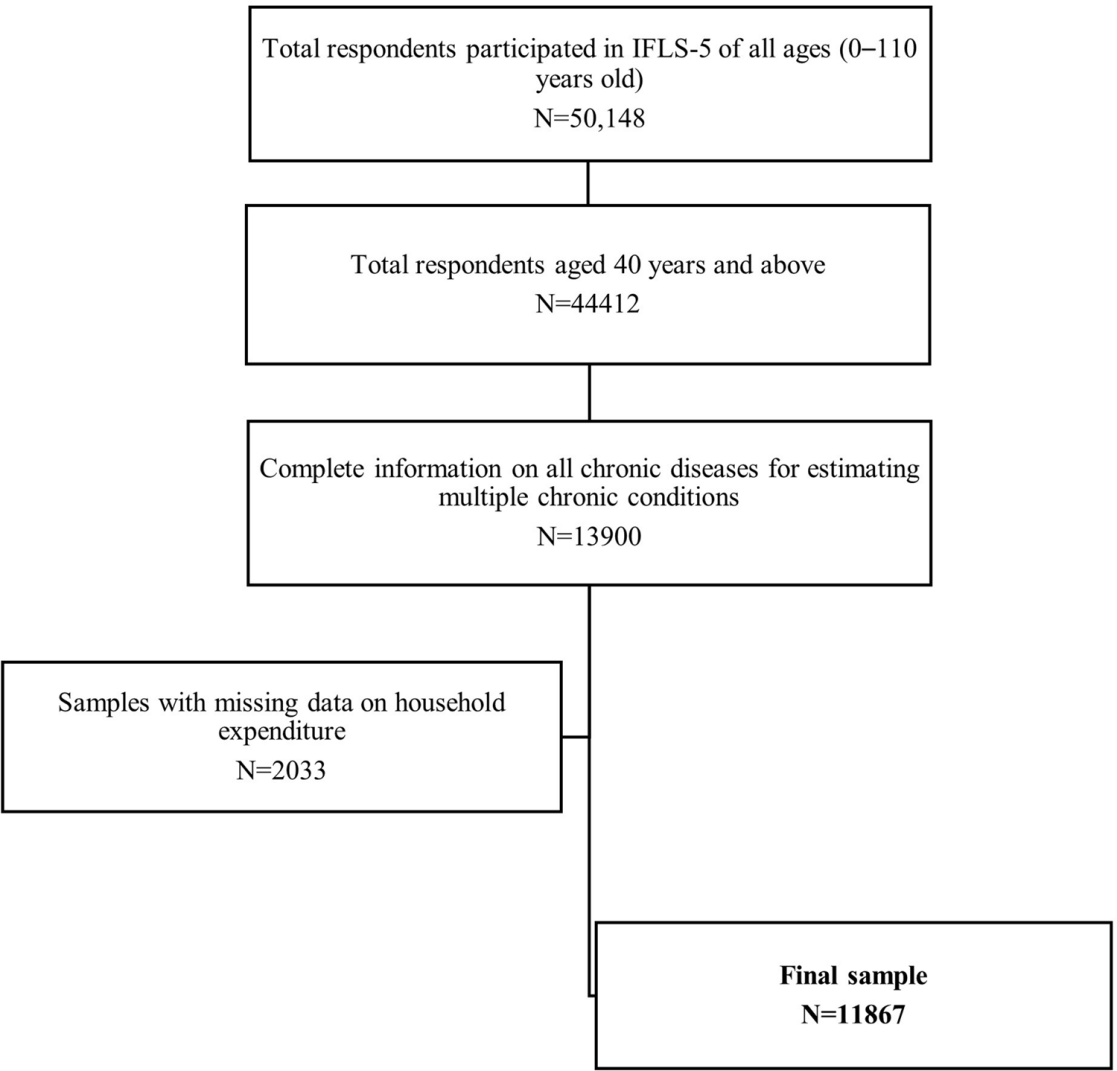
Selection of participants from IFLS-5.

Of 44,412 respondents aged 40 years or above interviewed in the survey, 30,512 (68.7%) respondents did not complete the question related to chronic diseases; thus, they were excluded from this study. We further excluded 2033 respondents (14.6%) whose household expenditure data were not available and surpassed the allowance limit for missing data (≤ 10% ± 5%). Figure 1 depicts a flow chart of the respondents' inclusion and reasons for exclusion. Other factors, such as physical activity, frequency of vegetable and fruit consumption, and BMI, also contained missing values but did not exceed the allowance limit [See additional file 1: Table S3, Supplementary data]. Hence, these data were still included in the analysis using the listwise deletion method^31^. As a result, 11,867 respondents were included, of which 68.3% were from the middle-aged population (${\overline{\text{x}}}$  = 49.0; SD = 43.4–54.6) and 31.7% were from the elderly population (${\overline{\text{x}}}$ = 70.0; SD = 52.9–87.0).

Most of the included respondents were middle-aged (68.3%), female (53.9%), living in urban areas (56.9%), graduated from elementary school (47.1%), had worked in the last 12 months (71.3%), currently married (77.0%), classified into the fourth quintile of economic status (20.1%), vigorously active (38.1%), and non-smoker (60.2%) as shown in Table 1 below.

**Table 1:** Sociodemographic characteristics, health-related behaviors, and multimorbidity of the middle-aged and elderly population in Indonesia.

Variable	40–59 years (${\overline{\text{x}}}$ = 49 years) N = 8102 (68.3%)	≥ 60 years (${\overline{\text{x}}}$ = 70 years) N = 3765 (31.7%)	*p*-value	All respondents (N = 11,867)
Sociodemographic characteristics				
Gender				
Male	3753 (46.3%)	1709 (45.4%)	0.344	5462 (46.1%)
Female	4349 (53.7%)	2056 (54.6%)		6405 (53.9%)
Place of living				
Urban	4728 (58.4%)	2025 (53.8%)	< 0.001	6753 (56.9%)
Rural	3374 (41.6%)	1740 (46.2%)		5114 (43.1%)
Educational level				
Kindergarten or less	576 (7.1% )	939 (24.9% )	< 0.001	1515 (12.8%)
Elementary school	3668 (45.3%)	1922 (51% )		5590 (47.1%)
Middle school	1208 (14.9%)	321 (8.5%)		1529 (12.9%)
High school	1771 (21.9%)	329 (8.7%)		2100 (17.7%)
Graduate or above	839 (10.4%)	180 (4.8%)		1019 (8.6%)
Cannot be classified	40 (0.5%)	74 (2.0%)		114 (0.9%)
Working status				
Working	6588 (81.3%)	1875 (49.8%)	< 0.001	8463 (71.3%)
Not working	1514 (18.7%)	1890 (50.2%)		3404 (28.7%)
Marital status				
Unmarried	1158 (14.8%)	1568 (41.6%)	< 0.001	2726 (23.0%)
Married	6944 (85.7%)	2197 (58.4%)		9141 (77.0%)
Economic status				
Q1 (lowest)	1543 (19.0%)	831 (22.1%)	< 0.001	2374 (20.0%)
Q2	1625 (20.1%)	751 (19.9%)		2376 (20.0%)
Q3	1676 (20.7%)	695 (18.5%)		2371 (20.0%)
Q4	1680 (20.7%)	705 (18.7%)		2385 (20.1%)
Q5 (highest)	1578 (19.5%)	783 (20.8%)		2361 (19.9%)
Health-related behavior				
Physical activity				
Mild	2496 (30.8%)	1072 (28.5%)	< 0.001	3568 (30.1%)
Moderate	1775 (21.9%)	566 (15.0%)		2341 (19.7%)
Vigorous	3371 (41.6%)	1147 (30.5%)		4518 (38.1%)
Missing data	460 (5.7%)	980 (26.0%)		1440 (12.1%)
Smoking habit				
Non-smoker	4988 (61.6%)	2152 (57.2%)	< 0.001	7140 (60.2%)
Former smoker	464 (5.7%)	504 (13.4%)		968 (8.1%)
Smoker	2650 (32.7%)	1109 (29.5%)		3759 (31.7%)
Fruits consumption frequency				
7 days per week	1581 (19.5%)	663 (17.6%)	< 0.001	2244 (18.9%)
4–6 days per week	1187 (14.7%)	452 (12.0%)		1639 (13.8%)
< 4 days per week	4867 (60.1%)	1666 (44.2%)		6533 (55.1%)
Missing data	467 (5.8%)	984 (26.1%)		1451 (12.2%)
Vegetable consumption frequency				
7 days per week	3215 (39.7%)	1119 (29.7%)	0.002	4334 (36.5%)
4–6 days per week	1195 (14.7%)	385 (10.2%)		1580 (13.3%)
< 4 days per week	3225 (39.8%)	1277 (33.9%)		4502 (38.0%)
Missing data	467 (5.8%)	984 (26.1%)		1451 (12.2%)
Body mass index				
Underweight	552 (6.8%)	700 (18.6%)	< 0.001	1252 (10.6%)
Normal	2617 (32.3%)	1431 (38.0%)		4048 (34.0%)
Overweight	1349 (16.7%)	502 (13.3%)		1851 (15.6%)
Obese	3253 (40.2%)	777 (20.6%)		4030 (34.0%)
Missing data	331 (4.1%)	355 (9.4%)		686 (5.8%)
Multimorbidity				
Yes	1267 (15.6%)	937 (24.9%)	< 0.001	2204 (18.6%)
No	6835 (84.4%)	2828 (75.1%)		9663 (81.4%)

### Prevalence and patterns of multimorbidity

The prevalence of multimorbidity by age-groups was 15.6% in the middle-aged population and 24.9% in the elderly population (Table 1). Among 2204 respondents who had multimorbidity, 57.5% of them were middle-aged population and 42.5% were elderly (Table 2).

**Table 2:** Prevalence of chronic diseases and multimorbidity.

	Prevalence (N = 11,867)							
	40–59 years N = 8102 (68.3%)		≥ 60 years N = 3765 (31.7%)		*p*-value	N (%)	95% CI	Total
	Male N = 3753 (31.6%)	Female N = 4349 (36.6%)	Male N = 1709 (14.4%)	Female N = 2056 (17.3%)				
Multimorbidity	417 (18.9%)	850 (38.6%)	383 (17.4%)	554 (25.1%)	< 0.001^a^	18.6	17.87 to 19.27	2204
Multimorbidity (by age group)	1267 (57.5%)		937 (42.5%)					
Chronic diseases								
Hypertension	484 (17.6%)	1088 (39.6%)	436 (15.8%)	742 (27.0%)	< 0.001^a^	23.2	22.41 to 23.93	2750
Diabetes mellitus	151 (25.3%)	210 (35.2%)	117 (19.6%)	119 (19.9%)	< 0.001^a^	5.0	4.64 to 5.42	597
Asthma	80 (22.4%)	121 (33.9%)	74 (20.7%)	82 (23.0%)	< 0.001^a^	3.0	2.7 to 3.32	357
Chronic lung diseases	72 (29.6%)	74 (30.5%)	53 (21.8%)	44 (18.1%)	0.002^a^	2.0	1.79 to 2.3	243
Cardiac diseases	66 (17.7%)	135 (36.2%)	79 (21.2%)	93 (24.9%)	< 0.001^a^	3.1	2.83 to 3.46	373
Liver diseases	47 (41.6%)	28 (24.8%)	25 (22.1%)	13 (11.5%)	0.390	1.0	0.78 to 1.13	113
Stroke	45 (17.2%)	57 (21.7%)	82 (31.3%)	78 (29.8%)	< 0.001^a^	2.2	1.94 to 2.47	262
Cancer/malignancies	9 (9.0%)	63 (63.0%)	12 (12.0%)	16 (16.0%)	0.621	0.8	0.68 to 1.01	100
Arthritis/gout	232 (18.5%)	479 (38.3%)	185 (14.8%)	355 (28.4%)	< 0.001^a^	10.5	9.99 to 11.09	1251
Hypercholesterolemia	235 (25.8%)	416 (45.6%)	104 (11.4%)	157 (17.2%)	< 0.001^a^	7.7	7.2 to − 8.16	912
Prostate illness	21 (20.4%)	–	82 (79.6%)	–	< 0.001^a^	0.9	0.7 to 1.03	103
Kidney diseases	94 (39.0%)	84 (34.9%)	37 (15.3%)	26 (10.8%)	0.125	2.0	1.78 to 2.28	241
Digestive diseases	343 (24.5%)	626 (44.7%)	151 (10.8%)	280 (20.0%)	0.135	11.8	11.22 to 12.38	1400
Psychiatric problem	12 (30.0%)	17 (42.5%)	5 (12.5%)	6 (15.0%)	0.534	0.3	0.23 to 0.44	40
Memory-related diseases	11 (25.0%)	9 (20.5%)	10 (22.7%)	14 (31.8%)	0.004^a^	0.4	0.26 to 0.48	44

^a^p-value < 0.05 at the 5% level of significance.

Multimorbidity was more likely to be higher in females in both populations of which 38.6% were middle-aged and 25.1% were elderly (Table 2). Moreover, compared to the population with multimorbidity, the prevalence of multimorbidity also tended to be higher in populations who were living in urban areas, had a primary educational level, were actively working, were currently married, had the highest economic status, mild physically active, were non-smoker, had the least consumption of fruits, had the most frequent consumption of vegetable, and population who were obese [See additional file: Table S2, Supplementary data].

Table 3 below lists the five most frequently occurring combinations of chronic diseases among respondents with multimorbidity^32^. Hypertension appeared as the most frequently occurring disease in all age and sex groups, followed by digestive diseases, arthritis, hypercholesterolemia, diabetes mellitus, heart diseases, and stroke. A combination of hypertension with hypercholesterolemia, arthritis, digestive diseases, and diabetes were the most commonly occurring dyads and triads in both male and female groups (Table 3).

**Table 3:** Leading five most commonly occurring combinations of multimorbidity in the study population.

Age	Gender	Order	Diseases dyads	Prevalence	95% CI	Diseases triads	Prevalence	95% CI
40–59 years	Male	1	Hypertension–hypercholesterolemia	0.9	0.76–1.11	Hypertension–arthritis–digestive diseases	0.2	0.15–0.32
		2	Hypertension–arthritis	0.7	0.57–0.88	Hypertension–hypercholesterolemia–digestive diseases	0.2	0.14–0.31
		3	Hypertension–digestive diseases	0.7	0.56–0.86	Hypertension–arthritis–hypercholesterolemia	0.2	0.12–0.28
		4	Hypertension–diabetes	0.4	0.28–0.51	Hypertension–diabetes–hypercholesterolemia	0.2	0.10–0.25
		5	Arthritis–digestive diseases	0.4	0.28–0.5	Hypertension–cardiac diseases–hypercholesterolemia	0.1	0.07–0.20
	Female	1	Hypertension–digestive diseases	1.7	1.43–1.89	Hypertension–arthritis–hypercholesterolemia	0.4	0.30–0.54
		2	Hypertension–hypercholesterolemia	1.6	1.41–1.87	Hypertension–arthritis–digestive diseases	0.4	0.30–0.53
		3	Hypertension–arthritis	1.6	1.34–1.79	Hypertension–Hypercholesterolemia–digestive diseases	0.4	0.29–0.52
		4	Arthritis–digestive diseases	1.0	0.78–1.13	Hypertension–diabetes–hypercholesterolemia	0.3	0.24–0.45
		5	Hypertension–diabetes	0.9	0.77–1.12	Arthritis–hypercholesterolemia–digestive diseases	0.2	0.13–0.30
≥ 60 years	Male	1	Hypertension–arthritis	0.6	0.5–0.78	Hypertension–diabetes–hypercholesterolemia	0.2	0.09–0.23
		2	Hypertension–hypercholesterolemia	0.5	0.39–0.64	Hypertension–stroke–hypercholesterolemia	0.1	0.06–0.18
		3	Hypertension–diabetes	0.5	0.36–0.61	Hypertension–arthritis–digestive diseases	0.1	0.06–0.18
		4	Hypertension–digestive diseases	0.5	0.35–0.6	Hypertension–cardiac diseases–hypercholesterolemia	0.1	0.05–0.17
		5	Hypertension–stroke	0.4	0.32–0.56	Hypertension–cardiac diseases–digestive diseases and Hypertension–arthritis–hypercholesterolemia	0.1	0.04–0.16
	Female	1	Hypertension–arthritis	1.6	1.4–1.85	Hypertension–arthritis–digestive diseases	0.4	0.32–0.56
		2	Hypertension–digestive diseases	1.1	0.95–1.34	Hypertension–arthritis–hypercholesterolemia	0.3	0.17–0.35
		3	Hypertension–hypercholesterolemia	0.8	0.68–1.01	Hypertension–diabetes–hypercholesterolemia	0.2	0.12–0.28
		4	Arthritis–digestive diseases	0.7	0.54–0.84	Hypertension–hypercholesterolemia–digestive diseases	0.2	0.12–0.28
		5	Hypertension–diabetes	0.6	0.47–0.76	Hypertension–diabetes–digestive diseases	0.2	0.09–0.24

### Factors associated with multimorbidity

**Figure 2:**
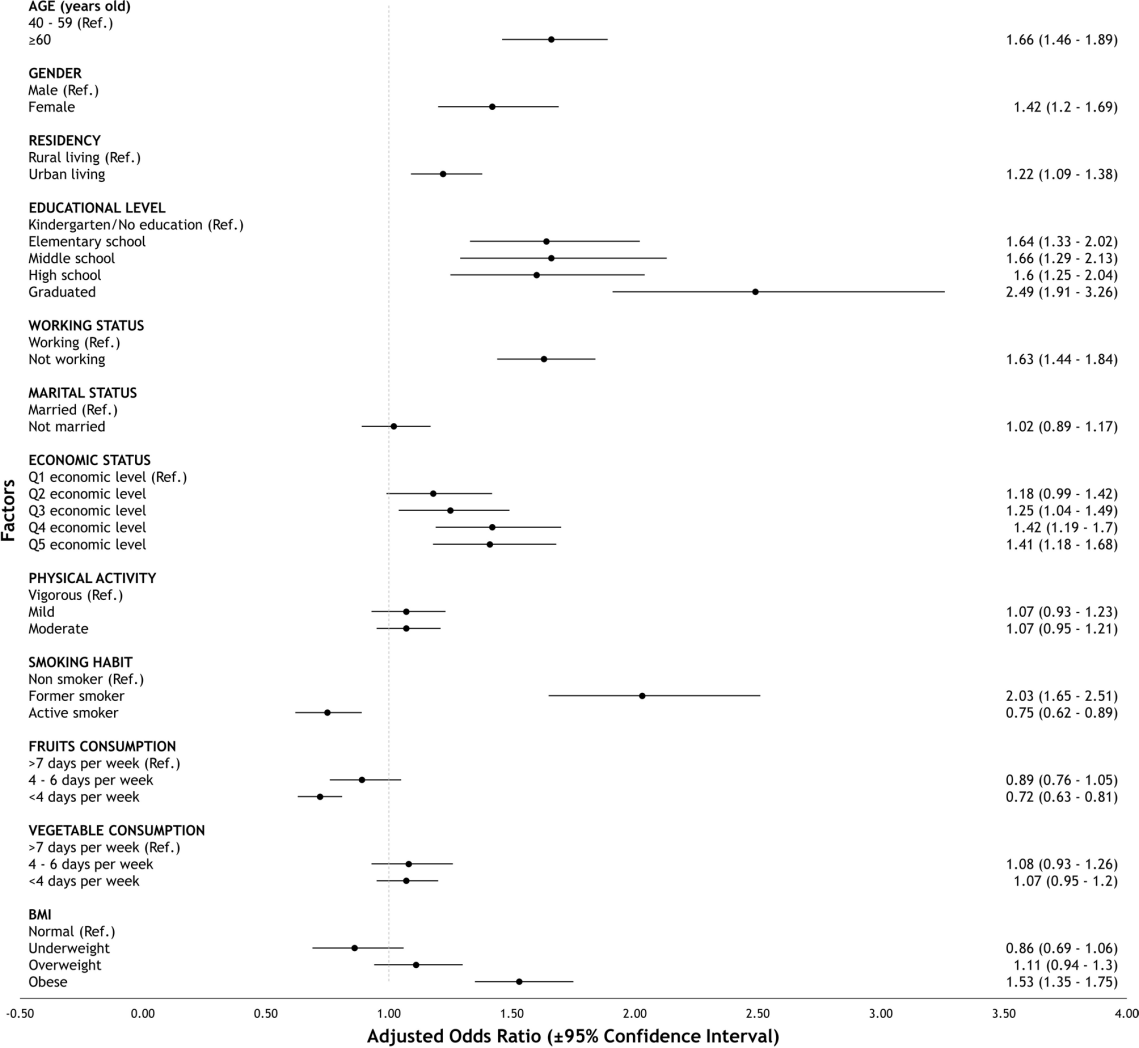
Forest plot of factors associated with multimorbidity in Indonesia.

**Figure 3:**
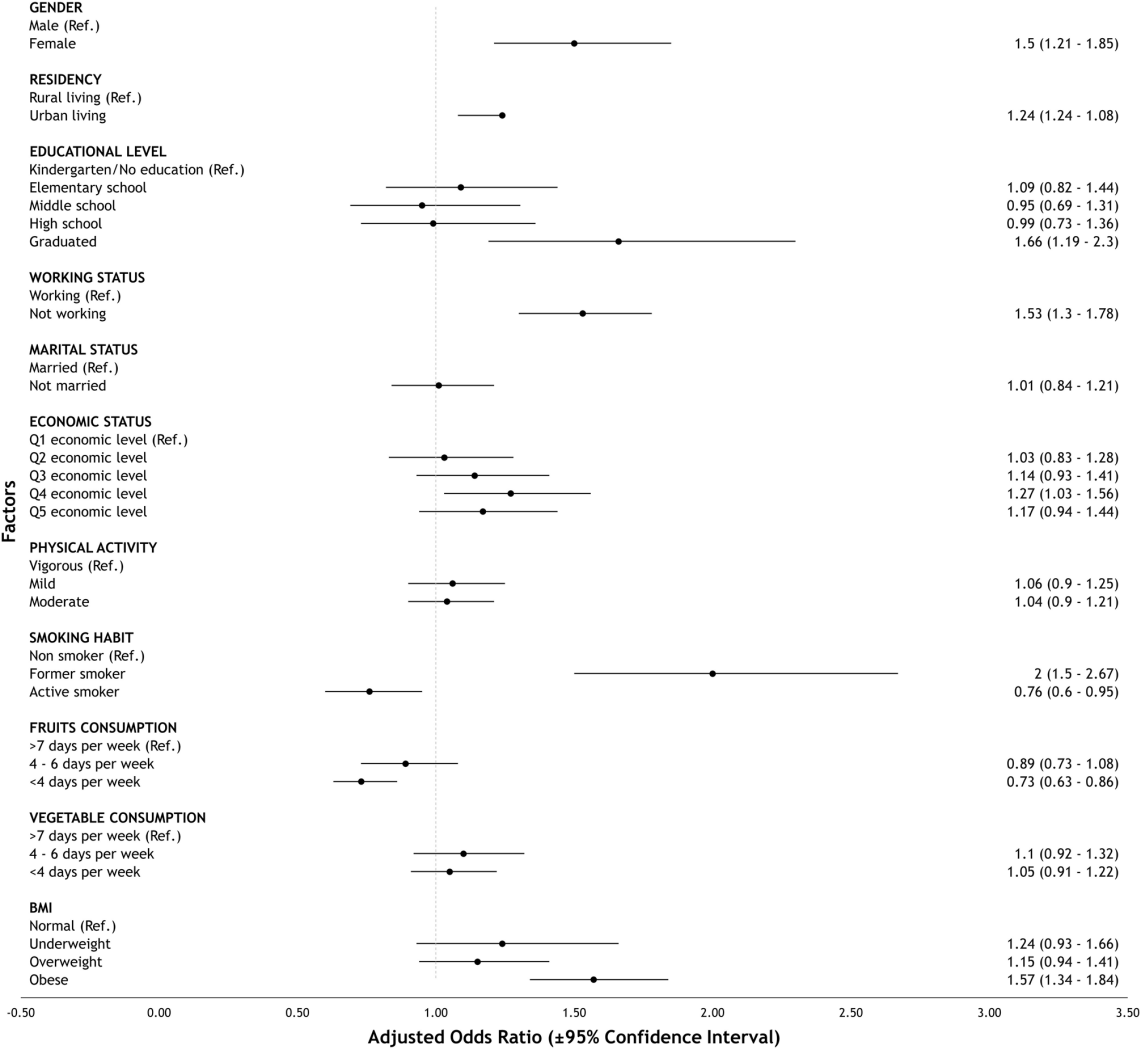
Forest plot of factors associated with multimorbidity in middle-aged population in Indonesia.

**Figure 4:**
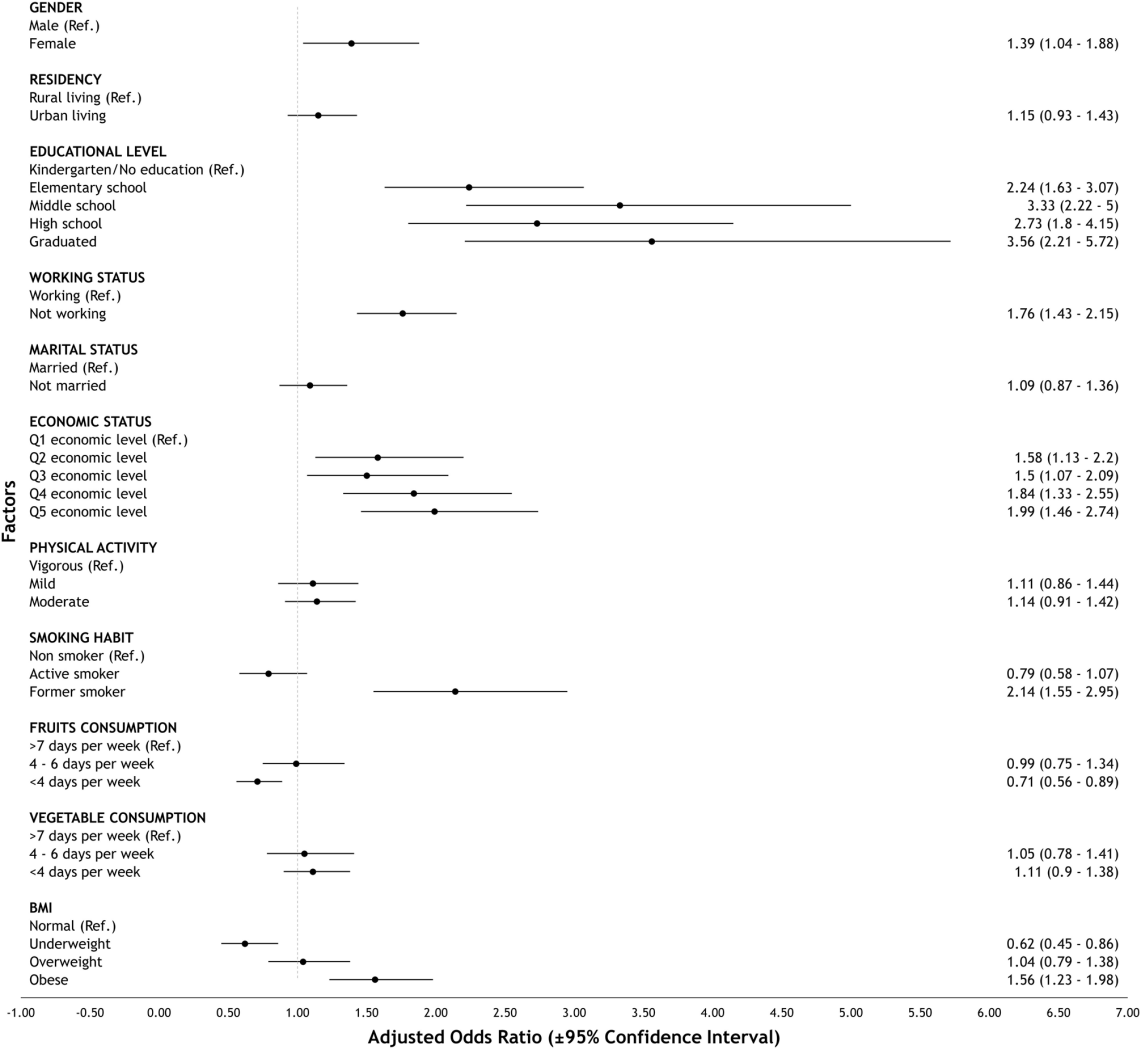
Forest plot of factors associated with multimorbidity in elderly population in Indonesia.

Based on bivariate analyses, all sociodemographic and health-related behavioral factors were selected as potential factors associated with multimorbidity (p < 0.25)^29,30^ [See additional file: Table S2, Supplementary data]. Furthermore, all factors included in the multivariable model exhibited no multicollinearity by having a value of VIF < 10. In the multivariate model, being elderly (AOR: 1.66; 95% CI 1.46–1.89), female (AOR: 1.42; 95% CI 1.20–1.69), those who lived in urban areas (AOR: 1.22; 95% CI 1.09–1.38), had a high educational level (AOR: 2.49; 95% CI 1.91–3.26), did not work in the last 12 months (AOR: 1.63; 95% CI 1.44–1.84), had a high economic level (AOR: 1.41; 95% CI 1.18–1.68), former smokers (AOR: 2.03; 95% CI 1.65–2.51), and those with obesity (AOR: 1.53; 95% CI 1.35–1.75) showed a significant association with multimorbidity. Those who had a frequency of less than 4 days per week of fruit consumption (AOR: 0.72; 95% CI 0.63–0.81) had a lower likelihood of developing multimorbidity (Table 4). ﻿ Fig. 2 shows a forest plot of associations between sociodemographic and health-related behavioral factors with multimorbidity in Indonesia. The results of the Omnibus test showed p < 0.001, indicating that the multivariable analysis model was feasible and fulfilled the meaning of the model. Furthermore, the results of the Nagelkerke R-Square test indicated that the factors in the model explained the multimorbidity by 10.3%. Furthermore, we conducted a subgroup analysis that is available in Table 5 below with the visualization through the forest plot model as shown in Figs. 3 and 4. The subgroup analysis result showed mostly similar point estimates for both age groups but not all remained significant.

**Table 4:** Factors associated with multimorbidity in Indonesia.

Factors	Multimorbidity^b^			
	p-value	AOR	CI (95%)	
			Lower	Upper
Age (years)				
40–59	Ref			
≥ 60	< 0.001^a^	1.66	1.46	1.89
Gender				
Male	Ref			
Female	< 0.001^a^	1.42	1.20	1.69
Location of living				
Rural	Ref			
Urban	< 0.001^a^	1.22	1.09	1.38
Educational level				
Kindergarten or without formal education	Ref			
Elementary school	< 0.001^a^	1.64	1.33	2.02
Middle school	< 0.001^a^	1.66	1.29	2.13
High school	< 0.001^a^	1.60	1.25	2.04
Graduated or above	< 0.001^a^	2.49	1.91	3.26
Working status				
Working	Ref			
Not working	< 0.001^a^	1.63	1.44	1.84
Marital status				
Married	Ref			
Not married	0.761	1.02	0.89	1.17
Economic status				
Q (lowest)	Ref			
Q2	0.071	1.18	0.99	1.42
Q3	0.016^a^	1.25	1.04	1.49
Q4	< 0.001^a^	1.42	1.19	1.70
Q5 (highest)	< 0.001^a^	1.41	1.18	1.68
Physical activity				
Vigorous	Ref			
Mild	0.323	1.07	0.93	1.23
Moderate	0.270	1.07	0.95	1.21
Smoking habit				
Non-smoker	Ref			
Former smoker	< 0.001^a^	2.03	1.65	2.51
Smoker	0.001^a^	0.75	0.62	0.89
Fruits consumption frequency				
7 days per week	Ref			
4–6 days per week	0.179	0.89	0.76	1.05
< 4 days per week	< 0.001^a^	0.72	0.63	0.81
Vegetable consumption frequency				
7 days per week	Ref			
4–6 days per week	0.322	1.08	0.93	1.26
< 4 days per week	0.284	1.07	0.95	1.20
Body mass index				
Normal	Ref			
Underweight	0.158	0.86	0.69	1.06
Overweight	0.229	1.11	0.94	1.30
Obese	< 0.001^a^	1.53	1.35	1.75

^a^Significant factor (p < 0.05).

^b^Nagelkerke R-square test: 10.3%; Omnibus test: p < 0.001.

*AOR* adjusted odds ratios, *CI* confidence intervals.

**Table 5:** Subgroup analysis of factors associated with multimorbidity in Indonesia based on age group.

Factors	Multimorbidity (40–59 years)^b^				Multimorbidity (≥ 60 years)^c^			
	p-value	AOR	95% CI		p-value	AOR	95% CI	
			Lower	Upper			Lower	Upper
Gender								
Male	Ref				Ref			
Female	< 0.001^a^	1.50	1.21	1.85	0.029^a^	1.39	1.04	1.88
Location of living								
Rural	Ref				Ref			
Urban	0.002^a^	1.24	1.08	1.43	0.184	1.15	0.93	1.43
Educational level								
Kindergarten or without formal education	Ref				Ref			
Elementary school	0.557	1.09	0.82	1.44	< 0.001^a^	2.24	1.63	3.07
Middle school	0.749	0.95	0.691	1.305	< 0.001^a^	3.33	2.22	5.00
High school	0.988	0.99	0.73	1.36	< 0.001^a^	2.73	1.80	4.15
Graduated or above	0.003^a^	1.66	1.19	2.30	< 0.001^a^	3.56	2.21	5.72
Working status								
Working	Ref				Ref			
Not working	< 0.001^a^	1.53	1.30	1.78	< 0.001^a^	1.76	1.43	2.15
Marital status								
Married	Ref				Ref			
Not married	0.940	1.01	0.84	1.21	0.453	1.09	0.87	1.36
Economic status								
Q (lowest)	Ref				Ref			
Q2	0.807	1.03	0.83	1.28	0.007^a^	1.58	1.13	2.20
Q3	0.214	1.14	0.93	1.41	0.019^a^	1.50	1.07	2.09
Q4	0.026^a^	1.27	1.03	1.56	< 0.001^a^	1.84	1.33	2.55
Q5 (highest)	0.162	1.17	0.94	1.44	< 0.001^a^	1.99	1.46	2.74
Physical activity								
Vigorous	Ref				Ref			
Mild	0.498	1.06	0.90	1.25	0.43	1.11	0.86	1.44
Moderate	0.571	1.04	0.9	1.21	0.25	1.14	0.91	1.42
Smoking habit								
Non-smoker	Ref				Ref			
Former smoker	< 0.001^a^	2.00	1.50	2.67	< 0.001^a^	2.14	1.55	2.95
Smoker	0.016^a^	0.76	0.60	0.95	0.121	0.79	0.58	1.07
Fruits consumption frequency								
7 days per week	Ref				Ref			
4–6 days per week	0.238	0.89	0.73	1.08	0.988	0.99	0.75	1.34
< 4 days per week	< 0.001^a^	0.73	0.63	0.86	0.003^a^	0.71	0.56	0.89
Vegetable consumption frequency								
7 days per week	Ref				Ref			
4–6 days per week	0.303	1.10	0.92	1.32	0.749	1.05	0.78	1.41
< 4 days per week	0.476	1.05	0.91	1.22	0.332	1.11	0.90	1.38
Body mass index								
Normal	Ref				Ref			
Underweight	0.141	1.24	0.93	1.66	0.004^a^	0.62	0.45	0.86
Overweight	0.171	1.15	0.94	1.41	0.768	1.04	0.79	1.38
Obese	< 0.001^a^	1.57	1.34	1.84	< 0.001^a^	1.56	1.23	1.98

*AOR* adjusted odds ratios, *CI* confidence intervals.

^a^Significant factor (p < 0.05).

^b^Nagelkerke R-square test: 7.1%; Omnibus test: p < 0.001.

^c^Nagelkerke R-square test: 17.1%; Omnibus test: p < 0.001.

## Discussion

This study assessed multimorbidity prevalence in the Indonesian middle-aged and elderly population using national data from the IFLS-5. About one-sixth of the middle-aged population and a quarter of the elderly population experienced multimorbidity, but the pattern varied markedly across different sociodemographic and health-related behavioral characteristics. The most commonly occurring multimorbidities were hypertension, arthritis, hypercholesterolemia, digestive diseases, diabetes mellitus, and cardiac diseases. Increasing age, female gender, urban living, high educational level, unemployed, high per-capita household expenditure, smoking habit, and obesity were significantly associated with multimorbidity.

We observed that most of the combinations of multimorbidity such as hypertension, arthritis, diabetes, and cardiovascular disease in our study were similar to the most prevalent chronic diseases in Indonesia in 2018^32^. We further observed that hypertension was the most commonly reported disease in all combinations of multimorbidity and among all age groups. Hypertension is commonly associated with multimorbidity and most patients with hypertension have at least one other chronic condition^33^. Hypertension is mostly concurring with other chronic diseases due to its metabolical pathway that could lead to another metabolic impairment^34,35^. Proper prevention of hypertension can improve the health status of most of the population and reduce the risk of developing multimorbidity^36^. Our findings showed that the combinations of hypertension with cardiovascular diseases, stroke, digestive diseases, hyperlipidemia, diabetes, and arthritis were common in multimorbidity among all age groups and gender, the difference was only the order of the most prevalent combinations among the age groups. Overall, the prevalence of cardiovascular diseases and stroke was mainly observed in males than in females. Some studies have reported the same finding concerning the higher prevalence of stroke in men than women^37,38^. The rate of stroke incidence per 10,000 people each year was also reported to be remarkably higher in men (13.96) than in women (8.66)^39^. It is also shown from this study that stroke was more likely to happen in the elderly population than in the middle-aged. This finding is in line with another study that reported a rise in stroke incidents in normotensive elderly men (23.9/1000) than in middle-aged men (2.7/1000)^40^. The risk to experience stroke is two to seven times higher among patients with hypertension^40^. Meanwhile, the most prevalent combination of diseases that were found in females was digestive diseases combined with another chronic condition, particularly hypertension. Females tend to have a higher risk of developing digestive problems than men^41^. Additionally, the state of high blood pressure correlates with inflammation and increment of the gastrointestinal epithelial barrier permeability^42^. It has been also reported that there is an association between microbiological changes in the gastrointestinal system and hypertension^42^.

A combination of diabetes, hyperlipidemia, and hypertension was also highly observed both in males and females among all age groups. Hyperinsulinemia condition in patients with diabetes is associated with impaired blood vessels due to oxidative pressure and inflammation influenced by the high amount of insulin level in the blood. Therefore, this condition may induce impaired vasodilatation function of the blood vessels resulting in high blood pressure^43^, while hyperlipidemia condition may impair endothelial function which disrupts the production of nitric oxide and affects the regulation of blood pressure^44^. Studies have shown that the anti-oxidant and anti-inflammatory function of high-dense lipoprotein (HDL) in patients with diabetes is reduced, thus, increasing the risk for patients with diabetes to experience dyslipidemia^45,46^.

Another chronic disease that was frequently observed in the combination of chronic diseases is arthritis. A study showed that metabolic problem including hypertension, hyperglycemia, and hypercholesterolemia has a significant effect on arthritis development^47^. Osteoarthritis and hypertension shared traditional risk factors that might be associated with the combination of these two chronic diseases^47^.

The most prevalent combination of chronic diseases was related to metabolic problems. This could be due to most chronic diseases sharing the same pathologic pathways^43,44^. Regardless, patients with mental health disorders have a higher risk of chronic disease development^48^ such as dyslipidemia, obesity, and cardiovascular diseases^49^. Our finding emphasizes the need for a proper definition of multimorbidity which is not only the existence of two or more chronic diseases but also the clinically related pattern that connects one chronic disease to other diseases.

We further observed that several sociodemographic and health-related behaviors are significantly associated with multimorbidity. Increasing age has a significant effect on multimorbidity development. This result corroborates with previous studies that showed an increment of multimorbidity cases in the aging population^50–52^. This finding is likely to be associated with physical and functional vulnerabilities in the aging population^52^. Studies showed that loss of physical and functional health due to aging could give rise to multimorbidity^52,53^. Inflammation, mitochondrial dysfunction, epigenetic alteration, hearing function, vision, and muscle strength all deteriorate as people age^3,54,55^. In this study, females tended to experience multimorbidity (63.7%) more than men (36.3%). This finding is also in line with previously published studies that showed the increased frequency of multimorbidity among women^3,14,15,56^. A potential reason for this finding could be women’s relatively higher tendency to use healthcare facilities^57,58^ and share their health conditions^59^. It is also possible that women are more likely to get more exposed to some risk factors of chronic diseases^60^. However, larger studies with more robust methodologies are needed to confirm our findings regarding gender differences contributing to multimorbidity.

Urban living is also significantly associated with multimorbidity development. The relatively high rate of air, noise, and light pollution in the urban region might lead to the development of asthma, allergy, cardiac diseases, and cancer^61^. Cities account for 78% of carbon emissions globally^62^, which have become triggers of inflammation and oxidative stress on the lungs. The increase in SO_2_, O_3_, and NO_2_ levels in the air correlates with the early onset of inflammatory bowel diseases (IBD)^63^ and asthma^64^. The low rate of outdoor activity in urban living leads to vitamin D deficiency; hence, it is associated with cardiovascular diseases, IBD, cancer, and asthma^61^. However, the association between urban living and multimorbidity among the elderly group was lost in the subgroup analysis. ﻿This may be due to the inclusion of a smaller number of patients, since most point estimates did not change much but the confidence intervals became larger.

Higher educational levels showed a significant association with multimorbidity. Educational level correlates with working status and has been reported as a significant factor associated with multimorbidity. This finding is in line with the previous studies^14,15^. The higher prevalence of multimorbidity among the population with better education could be associated with higher self-awareness of personal health, thus, making healthcare more accessible^65^. This indicates that populations with higher levels of education tend to report more on their health status and get diagnosed earlier. A previous study has shown that Indonesians with better education backgrounds are wealthier than those with lower levels of education^66^ which could play a role in providing better self-healthcare.

Multimorbidity was also found to be higher in the unemployed population than in those who had worked in the past 12 months during the survey. This finding agrees with some published research^15,67,68^. A study reported an increment in the risk for early exit from paid employment among workers with multimorbidity^69^. Moreover, the condition of chronic illness, specifically, multimorbidity has been a paramount factor that reduces employment chances^67^. Unemployed people may lack time management and productivity as well as lack social contacts and social status, thus, might be associated with multimorbidity^70^.

Being at a high level of economic status was significantly associated with multimorbidity. In a low-income country, chronic diseases were usually found to be higher in populations with a high economic status due to the epidemiological transition that a low-income country is facing^14^. The low prevalence of multimorbidity in low economic groups could also be due to the low ability of respondents from low economic levels to access health facilities and get a correct diagnosis of their health status^71^. Groups with low economic status tend to have a smoking habit and consume more alcohol and less balanced food nutrition; meanwhile, groups with high economic status tend to be less physically active and consume higher levels of fat, salt, and processed foods^72^. ﻿However, the association between economic status and multimorbidity in the middle-aged group was lost in the subgroup analysis. Again, this may be due to the inclusion of a smaller number of patients, since most point estimates did not change much but the confidence intervals became larger.

Smoking habits had a significant association with multimorbidity. Smoking habits increase the risk of cardiovascular disorders and multimorbidity^73^. Systemic inflammation that comes as a result of smoking could be an initiator of morbidity in asthma^74^. In this study, the result showed that the prevalence of multimorbidity in the former-smoker population was higher than in the active-smoker population. Despite the negative impacts of smoking they both had, this result may also be due to the population with multimorbidity may have stopped smoking^75^, thus, resulting in a high number in prevalence. The lower prevalence of multimorbidity in active smokers than in nonsmokers may be due to the fact that some respondents from the nonsmoking population with multimorbidity were passive smokers, that is, victims of cigarette smoke exposure who have the same risk of developing asthma, lung cancer, and other respiratory disorders^76^.

Surprisingly, we observed that a lower frequency of fruit consumption was associated with a lower likelihood of multimorbidity. The results were contrary to the previous studies, which showed that greater consumption of fruits appears to lower the risk of multimorbidity and mortality^77,78^. Unfortunately, the cross-sectional study that we applied to this study can not help determine cause and effect and thus could be responsible for this contrary finding. Moreover, this contrary result could be associated with the inequality of the population, and the data collection did not sufficiently represent the pattern of fruit consumption of the respondents because this consumption data was based on consumption in the past week. This could also be associated with the low fruit consumption rate of the Indonesian people, which on average was only 59.04% of the minimum nutritional adequacy rate set by the WHO—150 g/capita/day^79^. Next to fruit consumption, the frequency of vegetable consumption was not significantly associated with multimorbidity. This could be explained by the habits of Indonesian people in processing vegetables by boiling or frying. Moreover, the habit of adding kitchen spices, such as sugar and salt, when cooking vegetables in Indonesian society tended to be more than the recommended daily intake level; hence, it could be a positive determinant of the emergence of chronic diseases^80^. After all, fruits and vegetables are still becoming inaccessible foods for Indonesian people, particularly the low-income population^81^.

BMI was a significant factor associated with multimorbidity. The overweight group had a higher likelihood of developing multimorbidity than the normal BMI group. Overweight and obesity increase the risk of serious health problems, including mortality, hypertension, dyslipidemia, diabetes, heart problems, stroke, and osteoarthritis^82^*.* Obesity is a major determinant of the cause of a series of chronic diseases due to its natural mechanism of excess secretion of adipokines and a major contributor to dysfunction of the metabolic system involving fat and glucose, which significantly affects the dysfunction of the heart, liver, lungs, endocrine, and reproductive functions. Furthermore, obesity also contributes to immune system dysfunction due to the excessive secretion of inflammatory adipokines, thus leading to various diseases related to immune system disorders and cancer^83^.

Overall, some factors were not significantly associated with multimorbidity, unlike previous studies. In particular, marital status was not considered to be a significant factor associated with multimorbidity. Being married did not affect the development of hypertension and was thought to be a significant factor associated with mortality in men^84^. The risk of cardiovascular diseases was found to be higher both in the population who are single and widowed^85^. Furthermore, physical activity was not significantly associated with multimorbidity. Being physically active scientifically proven could reduce the risk of chronic conditions, such as diabetes, cardiac diseases, cancer, depression, and anxiety, as well as dementia^86^. The insignificant finding might be due to the available data could not represent the respondents' overall level of physical activity. The frequency of vegetable consumption was not significantly associated with multimorbidity. This could be explained by the habits of Indonesian people in processing vegetables by boiling or frying. Moreover, the habit of adding kitchen spices, such as sugar and salt, when cooking vegetables in Indonesian society tended to be more than the recommended daily intake level; hence, it could be a positive determinant of the emergence of chronic diseases^80^.

A comprehensive care approach has been suggested as the most suitable strategy for managing patients with multimorbidity. However, the delivery of primary healthcare in Indonesia is mainly built around the management of single diseases; therefore, healthcare professionals should integrate more patient-specific factors when designing tailored interventions to manage multimorbidity in Indonesia.

To the best of our knowledge, this is the first study to use a population-based national survey in Indonesia to comprehensively assess the patterns and factors associated with multimorbidity in the middle-aged and elderly population. Nevertheless, this study has some limitations. First, the data on multimorbidity in this study were largely based on self-reports; thus, many respondents with multimorbidity may not have been reported comprehensively due to a misdiagnosed condition, which can only be enforced through expert examination. Moreover, recall bias was probable—when respondents were unable to remember actual events—and the possibility that respondents tended not to report their poor health status, thereby reducing the accuracy and comprehensiveness of the data. Second, this study was based on a cross-sectional study; hence, it is difficult to determine absolute causality*.* Third, the use of AORs as a measure of association between two variables tends to overestimate the effect compared with prevalence ratios^87^. Fourth, a stepwise approach with manual backward elimination was conducted to decide which variables to include in the model, which may be prone to chance findings. Future studies need to justify the choice of potential variables included in the model based on the literature. Fifth, our unweighted findings may not be fully representative of the population. Future studies need to take the sampling weights into account in the data analysis. Moreover, further studies using more advanced analysis to establish multimorbidity patterns such as cluster and principal components analyses are needed. Future studies are also recommended to incorporate information regarding multimorbidity patterns in populations with different socio-economic characteristics.

## Conclusion

The prevalence of multimorbidity in the middle-aged and elderly population in Indonesia is relatively high, particularly in the population with poor health behavior. Hypertension was the highest prevalence of concurrence in chronic diseases and was most commonly found in a combination of multimorbidity. Several sociodemographic characteristics (aging, female, urban living, higher educational level, actively working, higher economic level) and health-related behaviors (smoking habit, less fruit consumption, obesity) had a significant association with multimorbidity*.* Therefore, healthcare professionals should integrate more patient-specific factors when designing tailored interventions to manage multimorbidity in Indonesia.

### Supplementary Information

Supplementary Tables.

## Data Availability

The raw data supporting the conclusions of this article will be made available by the authors, without undue reservation.
