# Supplementary material for: Findings from the Indonesian family life survey on patterns and factors associated with multimorbidity
Source: Sci Rep. 2023 Oct 30;13:18607. doi: 10.1038/s41598-023-42603-2 (PMC10616186; doi:10.1038/s41598-023-42603-2)
Supplement: Supplementary file 1 — Supplementary Tables. [file 41598_2023_42603_MOESM1_ESM.docx]

**Supplementary Data**

Table S1 STROBE Statement: Checklist of items that should be included in reports of cross-sectional studies

|  | Item No | Recommendation | Page No |
| --- | --- | --- | --- |
| **Title and abstract** | 1 | (*a*) Indicate the study’s design with a commonly used term in the title or the abstract | 1-2 |
|  |  | (*b*) Provide in the abstract an informative and balanced summary of what was done and what was found | 2 |
| Introduction | | | |
| Background/rationale | 2 | Explain the scientific background and rationale for the investigation being reported | 2-3 |
| Objectives | 3 | State specific objectives, including any prespecified hypotheses | 3 |
| Methods | | | |
| Study design | 4 | Present key elements of study design early in the paper | 3 |
| Setting | 5 | Describe the setting, locations, and relevant dates, including periods of recruitment, exposure, follow-up, and data collection | 3 |
| Participants | 6 | (*a*) Give the eligibility criteria, and the sources and methods of selection of participants | 3 |
| Variables | 7 | Clearly define all outcomes, exposures, predictors, potential confounders, and effect modifiers. Give diagnostic criteria, if applicable | 3-4 |
| Data sources/ measurement | 8* | For each variable of interest, give sources of data and details of methods of assessment (measurement). Describe comparability of assessment methods if there is more than one group | 4 |
| Bias | 9 | Describe any efforts to address potential sources of bias | 4 |
| Study size | 10 | Explain how the study size was arrived at | 3 |
| Quantitative variables | 11 | Explain how quantitative variables were handled in the analyses. If applicable, describe which groupings were chosen and why | 3-4 |
| Statistical methods | 12 | (*a*) Describe all statistical methods, including those used to control for confounding | 4 |
|  |  | (*b*) Describe any methods used to examine subgroups and interactions | 4 |
|  |  | (*c*) Explain how missing data were addressed | 4 |
|  |  | (*d*) If applicable, describe analytical methods taking account of sampling strategy | NA |
|  |  | (*e*) Describe any sensitivity analyses | NA |
| Results | | | |
| Participants | 13* | (a) Report numbers of individuals at each stage of study—eg numbers potentially eligible, examined for eligibility, confirmed eligible, included in the study, completing follow-up, and analysed | 5 |
|  |  | (b) Give reasons for non-participation at each stage | NA |
|  |  | (c) Consider use of a flow diagram | NA |
| Descriptive data | 14* | (a) Give characteristics of study participants (eg demographic, clinical, social) and information on exposures and potential confounders | 5-8 |
|  |  | (b) Indicate number of participants with missing data for each variable of interest | 5 |
| Outcome data | 15* | Report numbers of outcome events or summary measures | 5, 8, 10,14, 20 |
| Main results | 16 | (*a*) Give unadjusted estimates and, if applicable, confounder-adjusted estimates and their precision (eg, 95% confidence interval). Make clear which confounders were adjusted for and why they were included | 5-20 |
|  |  | (*b*) Report category boundaries when continuous variables were categorized | 4-8, |
|  |  | (*c*) If relevant, consider translating estimates of relative risk into absolute risk for a meaningful time period | NA |
| Other analyses | 17 | Report other analyses done—eg analyses of subgroups and interactions, and sensitivity analyses | 16-19 |
| Discussion | | | |
| Key results | 18 | Summarise key results with reference to study objectives | 20, 23 |
| Limitations | 19 | Discuss limitations of the study, taking into account sources of potential bias or imprecision. Discuss both direction and magnitude of any potential bias | 23 |
| Interpretation | 20 | Give a cautious overall interpretation of results considering objectives, limitations, multiplicity of analyses, results from similar studies, and other relevant evidence | 20-22 |
| Generalisability | 21 | Discuss the generalisability (external validity) of the study results | 20-22 |
| Other information | | | |
| Funding | 22 | Give the source of funding and the role of the funders for the present study and, if applicable, for the original study on which the present article is based | 23 |

*Give information separately for exposed and unexposed groups.

Table S2. Bivariate association between sociodemographic characteristics and health-related behavior and multimorbidity

| Factors | | Multimorbidity | | | Total | *p-value*  (p<0.05) |
| --- | --- | --- | --- | --- | --- | --- |
|  |  | **No**  N = 9663 (81.4%) | **Yes**  N = 2204 (18.6%) | **%^a^** |  |  |
| Sociodemographic | | | |  |  |  |
| Age (years) | |  |  |  |  |  |
| 40 – 59 | | 6.835 (84.4%) | 1267 (15.6%) | 57.5% | 8102 | <0.001^b^ |
| ≥ 60 | | 2.828 (75.1%) | 937 (24.9%) | 42.5% | 3765 |  |
| Gender | |  |  |  |  |  |
| Male | | 4662 (85.4%) | 800 (14.6%) | 36.3% | 5462 | <0.001^b^ |
| Female | | 5001 (78.1%) | 1404 (21.9%) | 63.7% | 6405 |  |
| Location of living | |  |  |  |  |  |
| Urban | | 5328 (78.9%) | 1425 (21.1%) | 64.7% | 6753 | <0.001^b^ |
| Rural | | 4335 (84.8%) | 779 (15.2%) | 35.3% | 5114 |  |
| Education level | |  |  |  |  |  |
| Kindergarten or less | | 1284 (84.8%) | 231 (15.2%) | 10.6% | 1515 | <0.001^b^ |
| Elementary school | | 4558 (81.5%) | 1032 (18.5%) | 47.2% | 5590 |  |
| Middle school | | 1238 (81.0%) | 291 (19.0%) | 13.3% | 1529 |  |
| High school | | 1733 (82.5%) | 367 (17.5%) | 16.8% | 2100 |  |
| Graduate or above | | 754 (74.0%) | 265 (26.0%) | 12.1% | 1019 |  |
| Working status | |  |  |  |  |  |
| Working | | 7248 (85.6%) | 1215 (14.4%) | 55.1% | 8463 | <0.001^b^ |
| Not working | | 2415 (70.9%) | 989 (29.1%) | 44.9% | 3404 |  |
| Marital status | |  |  |  |  |  |
| Not married | | 2116 (77.6%) | 610 (22.4%) | 27.7% | 2726 | <0.001^b^ |
| Married | | 7547 (82.6%) | 1594 (17.4%) | 72.3% | 9141 |  |
| Economic status | |  |  |  |  |  |
| Q1 (lowest) | | 2034 (85.7%) | 340 (14.3%) | 15.4% | 2374 | <0.001^b^ |
| Q2 | | 1977 (83.2%) | 399 (16.8%) | 18.2% | 2376 |  |
| Q3 | | 1957 (82.5%) | 414 (17.5%) | 18.8% | 2371 |  |
| Q4 | | 1886 (79.1%) | 499 (20.9%) | 22.6% | 2385 |  |
| Q5 (highest) | | 1809 (76.6%) | 552 (23.4%) | 25.0% | 2361 |  |
|  | |  |  |  |  |  |
|  | ***Health-related behavior*** | | | | | |
| Physical activity | |  |  |  |  |  |
| Mild | | 2863 (80.2%) | 705 (19.8%) | 38.2% | 3568 | <0.001^b^ |
| Moderate | | 1904 (81.3%) | 437 (18.7%) | 23.7% | 2341 |  |
| Vigorous | | 3816 (84.5%) | 702 (15.5%) | 38.1% | 4518 |  |
| Smoking habit | |  |  |  |  |  |
| Non-smoker | | 5652 (79.2%) | 1488 (20.8%) | 67.5% | 7140 | <0.001^b^ |
| Former smoker | | 656 (67.8%) | 312 (32.2%) | 14.2% | 968 |  |
| Smoker | | 3355 (89.3%) | 404 (10.7%) | 18.3% | 3759 |  |
| Fruit consumption frequency | |  |  |  |  |  |
| 7 days per week | | 1736 (77.4%) | 508 (22.6%) | 27.6% | 2244 | <0.001^b^ |
| 4 – 6 days per week | | 1311 (80.0%) | 328 (20.0%) | 17.8% | 1639 |  |
| < 4 days per week | | 5530 (84.6%) | 1003 (15.4%) | 54.6% | 6533 |  |
| Vegetable consumption frequency | |  |  |  |  |  |
| 7 days per week | | 3548 (81.9%) | 786 (18.1%) | 42.7% | 4334 | 0.030^b^ |
| 4 – 6 days per week | | 1275 (80.7%) | 305 (19.3%) | 16.6% | 1580 |  |
| < 4 days per week | | 3754 (83.4%) | 748 (16.6%) | 40.7% | 4502 |  |
| Body Mass Index | |  |  |  |  |  |
| Underweight | | 1061 (84.7%) | 191 (15.3%) | 9.4% | 1252 | <0.001^b^ |
| Normal | | 3465 (85.6%) | 583 (14.4%) | 28.7% | 4048 |  |
| Overweight | | 1535 (82.9%) | 316 (17.1%) | 15.6% | 1851 |  |
| Obese | | 3090 (76.7%) | 940 (23.3%) | 46.3% | 4030 |  |

Note:

^a^ Percentage of multimorbidity among population with multimorbidity.

^b^ Included in the initial model of multivariable analysis.

Table S3. Missing data in variables.

| **Variable** | **Total missing data (%)** | **Allowance limit for missing data** | **Status** |
| --- | --- | --- | --- |
| Age | - | ≤10% ± 5% (23) | - |
| Gender | - |  | - |
| Education level | 0.9 (cannot be classified) |  | Included and classified into a new category. |
| Working status | - |  | - |
| Marital status | - |  | - |
| Household expenditure | 14.6 |  | Excluded |
| Physical activity | 11.7 |  | Included |
| Smoking habit | - |  | - |
| Fruits consumption | 11.8 |  | Included |
| Vegetable consumption | 11.8 |  | Included |
| BMI | 5.9 |  | Included |

BMI, Body Mass Index
